# Supplementary material for: Inflammatory factors and risk of lung adenocarcinoma: a Mendelian randomization study mediated by blood metabolites
Source: Front Endocrinol (Lausanne). 2024 Aug 27;15:1446863. doi: 10.3389/fendo.2024.1446863 (PMC11384989; doi:10.3389/fendo.2024.1446863)
Supplement: Supplementary file 1 [file DataSheet1.zip › supplementary files/S2/GCST90200875/forest.pdf]

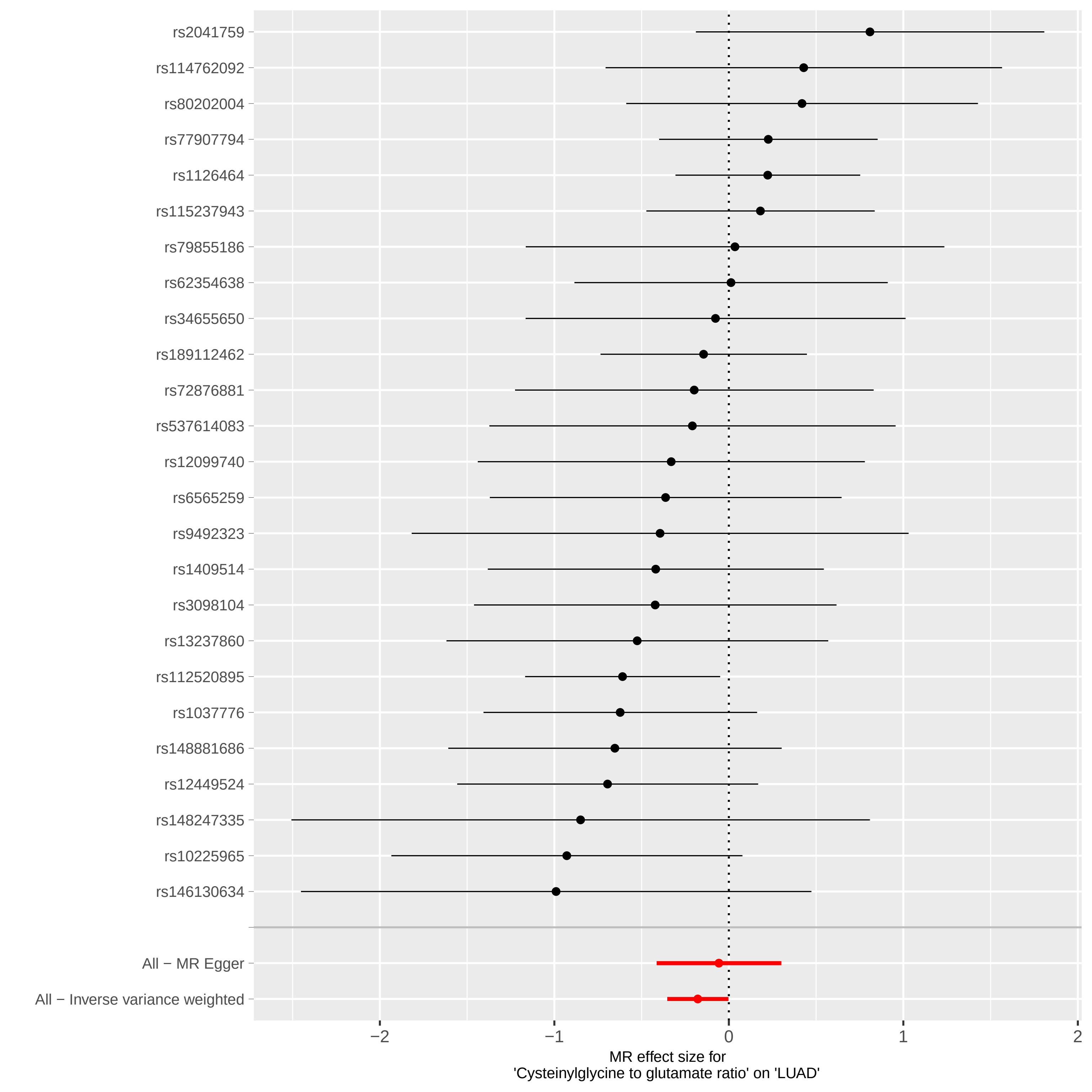
rs2041759
rs114762092
rs80202004
rs77907794
rs1126464
rs115237943
rs79855186
rs62354638
rs34655650
rs189112462
rs72876881
rs537614083
rs12099740
rs6565259
rs9492323
rs1409514
rs3098104
rs13237860
rs112520895
rs1037776
rs148881686
rs12449524
rs148247335
rs10225965
rs146130634
All − MR Egger
All − Inverse variance weighted
−2
−1
0
1
2
MR effect size for
'Cysteinylglycine to glutamate ratio' on 'LUAD'
